# Две модели развития инсулинорезистентности и стратегия борьбы с возрастзависимыми заболеваниями: обзор литературы

**DOI:** 10.14341/probl13090

**Published:** 2022-05-30

**Authors:** А. В. Мартюшев-Поклад, Д. С. Янкевич, М. В. Петрова, Н. Г. Савицкая

**Affiliations:** Федеральный научно-клинический центр реаниматологии и реабилитологии; Федеральный научно-клинический центр реаниматологии и реабилитологии; Федеральный научно-клинический центр реаниматологии и реабилитологии; Федеральный научно-клинический центр реаниматологии и реабилитологии

**Keywords:** инсулинорезистентность, гиперинсулинемия, ожирение, митохондриальная дисфункция, возрастзависимые заболевания, профилактика

## Abstract

**АКТУАЛЬНОСТЬ:**

АКТУАЛЬНОСТЬ. В основе многих возрастзависимых заболеваний (ВЗЗ), важнейшей проблемы современного здравоохранения, лежит инсулинорезистентность (ИР), поэтому адекватное понимание механизмов развития ИР необходимо для действенной профилактики ВЗЗ.

**ЦЕЛЬ:**

ЦЕЛЬ. Проанализировать существующие модели, отражающие причины и механизмы развития ИР, для обоснования наиболее результативной стратегии профилактики ВЗЗ.

**МАТЕРИАЛЫ И МЕТОДЫ:**

МАТЕРИАЛЫ И МЕТОДЫ. Анализ источников из elibrary.ru, PubMed и Google Scholar по ИР и гиперинсулинемии.

**РЕЗУЛЬТАТЫ:**

РЕЗУЛЬТАТЫ. В обзоре проанализированы две модели развития ИР и взаимосвязей между ИР, гиперинсулинемией (ГИ) и ожирением. Преобладающая модель рассматривает ожирение (нарушение баланса между калорийностью рациона и расходованием энергии) как основной фактор развития ИР, а ГИ — как следствие ИР, малозначимое для исходов ИР. Эта модель противоречит многим экспериментальным и клиническим данным. Соответствующая ей стратегия борьбы с ВЗЗ — гипокалорийный рацион и фармакотерапия ИР — показала недостаточную результативность.

Альтернативная модель (ИР как следствие ГИ, а ожирение — одно из проявлений ИР) лучше согласуется с современными экспериментальными и клиническими данными и позволяет более точно объяснять механизмы развития ВЗЗ и эффективнее корректировать неблагоприятные факторы образа жизни. Ей соответствует иная стратегия борьбы с ВЗЗ (акцент на низкоуглеводном рационе и достаточной пищевой паузе с учетом других факторов развития ИР).

**ЗАКЛЮЧЕНИЕ:**

ЗАКЛЮЧЕНИЕ. Пересмотр преобладающей модели развития ИР открывает возможности для повышения результативности ранней профилактики широкого спектра ВЗЗ, в которых значима роль ИР.

## Abstract (en)

**BACKGROUND:**

BACKGROUND: Insulin resistance (IR) is the root cause of most age-related diseases (ARD), the major challenge for today’s health systems. Therefore, adequate understanding of the mechanisms underlying IR is essential to build effective ARD prevention.

**OBJECTIVE:**

OBJECTIVE: Analyze the existing models of IR causation and progression in order to justify the most effective ARD prevention strategy.

**METHODS:**

METHODS: Search and analysis of publications on IR and hyperinsulinemia (HI) from databases elibrary.ru, PubMed, and Google Scholar.

**RESULTS:**

RESULTS: Two models of IR development are analyzed along with the relationship between IR, HI, and obesity. The prevailing model considers obesity (imbalance of caloric intake and energy expenditure) as the main factor in the development of IR; HI is seen as a consequence of IR, mostly insignificant for the outcomes of IR. The model contradicts many experimental and clinical findings. The strategy to combat ARDs that follows from the model (hypocaloric diet and pharmacotherapy of IR) has proven mostly ineffective.

The alternative model (IR as a consequence of HI, and obesity as one of IR manifestations) is more consistent with the pool of experimental and clinical data. It more precisely predicts ARD development and allows more adequate correction of adverse lifestyle factors. It corresponds to a different strategy for combating ARD: emphasis on low-carb diet and longer fasting window combined with consideration of other factors of IR.

**CONCLUSION:**

CONCLUSION: If the prevailing model of IR development is revised, this should open up opportunities for more effective early prevention of a wide range of chronic diseases in which the role of IR is significant.

## ВВЕДЕНИЕ

Возрастзависимые заболевания (ВЗЗ) — это ключевая проблема современной медицины. В последние десятилетия в мире наблюдается не только увеличение их частоты, что можно было бы связать со старением населения, но и «омоложение». Фармакотерапия, направленная на контроль отдельных проявлений ВЗЗ, практически не влияет на долгосрочный прогноз. Существует концепция, основанная на понимании тесной связи ВЗЗ с факторами образа жизни, и концепция о «предотвратимых смертях» от ВЗЗ. Доля ВЗЗ составляет 2/3 всей смертности граждан в возрасте до 75 лет [1][2].

Самым распространенным ранним проявлением ВЗЗ признана инсулинорезистентность (ИР). Так, ИР лежит в основе метаболического синдрома (МС) — признанного фактора риска сердечно-сосудистых заболеваний, злокачественных новообразований (ЗНО), сахарного диабета 2 типа (СД2), хронических обструктивных болезней легких, нейродегенеративных заболеваний, остеоартрита. ИР и МС тесно связаны с гиперинсулинемией (ГИ) и висцеральным ожирением.

Для эффективного противостояния эпидемии ВЗЗ необходимо глубокое и точное понимание ключевых патогенетических механизмов и причинно-следственных связей между факторами образа жизни и прогрессированием ВЗЗ. Это во многом определяет подход к профилактике ВЗЗ через коррекцию факторов образа жизни, а следовательно, и заболеваемость и смертность от ВЗЗ. Как показывают последние десятилетия, преобладающее представление о причинах и механизмах развития ИР и МС во многом не позволяет эффективно с ними бороться.

Цель — анализ существующих моделей, отражающих причины и механизмы развития ИР, для обоснования наиболее результативной стратегии профилактики ВЗЗ.

## МАТЕРИАЛЫ И МЕТОДЫ

С использованием ключевых слов “insulin resistance”, “hyperinsulinemia”, “age-related diseases” и их аналогов на русском языке была проанализирована литература из баз данных Научной электронной библиотеки elibrary.ru, PubMed и Google Scholar. Статьи выбирались на основе значимости для понимания роли ИР и ГИ, механизмов взаимосвязи между ИР, ГИ и ВЗЗ, а также для разработки практико-ориентированных подходов к модификации поведения пациента с ИР (ГИ), направленных на профилактику ВЗЗ и их осложнений.

Окончательный выбор источников был основан на суждении авторов о полноте и значимости для решения поставленных задач по выявлению подходов к борьбе с ВЗЗ.

Для анализа в данном обзоре были выбраны 49 статей, опубликованных в 1927–2021 гг. и позволяющих выявить ключевые параметры моделей развития ожирения и ИР, а также различия между моделями.

## РЕЗУЛЬТАТЫ

ИР определяют как снижение чувствительности клеток к инсулину и необходимость повышать выработку или дозу препаратов инсулина для достижения адекватного тканевого ответа. Существующие методы диагностики позволяют лишь качественно судить о наличии ИР, но не оценить ее количественно. На ИР косвенно указывают ряд клинических признаков и лабораторные показатели. В широкой практике используется индекс ИР (HOMA-IR), который рассчитывается на основе уровня инсулина и глюкозы в крови натощак.

Резистентность к инсулину (нарушение способности пациента с диабетом адекватно отвечать на инсулин) была впервые описана в литературе в 1927 г. [3].

«Отцом» концепции ИР как ключевого механизма развития СД2 считается Gerald Reaven [4]. В конце 1980-х гг. он четко сформулировал роль ИР и ГИ в развитии СД2, а также указал на причинно-следственную связь между ИР и атерогенной дислипидемией, ожирением, СД2, артериальной гипертензией и ишемической болезнью сердца. Позже к этому списку добавились деменция и некоторые виды ЗНО.

ИР — это элемент естественной адаптации организма к особым физиологическим условиям, на что указывает существование физиологической ИР. Ее смысл заключается в необходимости регулировать доступность энергетических субстратов для разных тканей организма и процессы запасания энергии в жировой ткани.

Примером нормальной ИР является период сна: самый низкий уровень утилизации глюкозы наблюдается во время медленной фазы сна [5].

При нормальной беременности в организме матери значительно (на 50–60%) ограничивается утилизация глюкозы — с тем, чтобы обеспечить адекватное снабжение энергией плода в случае недостатка пищи. Эта физиологическая ИР обеспечивается выработкой плацентарных гормонов: человеческого плацентарного гормона роста и плацентарного лактогена, а также адипокинов — цитокинов, вырабатываемых жировой тканью матери (лептин, адипонектин, фактор некроза опухоли альфа (ФНО-α), интерлейкин-6 (ИЛ-6) и др.) [6]. После родов действие плацентарных гормонов прекращается, что должно приводить к естественному исчезновению ИР. Однако если на организм матери действуют другие факторы, способствующие развитию ИР (прежде всего неоптимальный рацион и режим питания, низкая физическая активность, кишечный дисбиоз), то ИР сохраняется и после родов [7].

Для периода полового созревания также характерно развитие временной ИР, которая может быть связана с колебаниями уровня гормона роста и функционально сопряженного с ним инсулиноподобного фактора роста 1, имеющего с инсулином общую систему передачи сигнала [8]. У детей с фоновой ИР в пубертате развивается более выраженная ИР, которая нередко не исчезает после пубертата, а продолжает прогрессировать. С ИР в период пубертата связывают дебют или обострение акне. Таким образом, физиологическая и патологическая ИР могут представлять непрерывный спектр состояний.

Существует обоснованное мнение о том, что ИР является адаптивной реакцией организма на избыток инсулина и заключается в формировании особой функциональной системы, затрагивающей основные мишени инсулина — печень, мышечную и жировую ткани [9].

В литературе представлены две принципиально различающиеся модели, описывающие причинно-следственные связи между ИР, ГИ и висцеральным ожирением, а также механизм их естественного развития:

Согласно первой модели, первична избыточная масса тела (ожирение), вследствие которой развивается ИР, а ГИ является компенсаторной реакцией поджелудочной железы на ИР.

Вторая модель предполагает, что первична избыточная выработка инсулина (ГИ), вследствие которой развивается ИР и висцеральное ожирение.

## Модель 1: ИР как следствие ожирения и воспаления, ГИ как компенсаторная реакция на ИР.

Первая модель патогенеза ИР, сформировавшаяся в 1980-е гг. и в современном варианте подробно изложенная в обзоре 2018 г. [10], по-видимому, преобладает в российском профессиональном сообществе [11].

Согласно этой модели:

Таким образом, в рамках этой модели патогенеза феномен ИР рассматривается как адаптация к условиям избыточного поступления нутриентов. На уровне клетки избыток питания вызывает стрессовую реакцию, в том числе окислительный стресс в митохондриях. Физиологическое значение ИР — снизить утилизацию глюкозы и усилить процессы анаболизма. Второй аспект ИР как адаптивной реакции: в период избытка энергии запасание ее в форме жиров.

ИР как снижение клеточного ответа на инсулин складывается из снижения количества рецепторов инсулина на поверхности клетки и нарушения трансдукции сигнала («пострецепторного дефекта»).

Основное звено запуска ИР — это избыточное по отношению к тратам поступление в организм питательных веществ (источников энергии), «избыточное питание». Нарушения обмена, связанные с инсулином, зависят от полноты утилизации поступивших энергетических субстратов (активности расходования аденозинтрифосфата): при недостаточной утилизации не происходит полного окисления жиров, и в клетке накапливаются активные формы кислорода (АФК), токсичные полупродукты окисления. В результате либо повышается уровень токсичных метаболитов (жиров — диацилглицеридов, керамидов, ацелкарнитина; углеводов — конечных продуктов гликирования), которые создают перегрузку процессов утилизации (вызывают стресс эндоплазматического ретикулума, окислительный стресс); либо в ответ на действие токсичных метаболитов питательных веществ развивается системное воспаление.

Ключевым механизмом развития ИР и, следовательно, связанных с ИР хронических заболеваний признается митохондриальная дисфункция [10], которая способствует эктопическому накоплению липидов и их метаболитов.

В норме на скелетные мышцы приходится до 80% расходов полученной с пищей глюкозы [10], поэтому недостаточная физическая активность служит важной причиной дисбаланса поступления и расходования энергии.

Важную роль в развитии ИР играют воспалительные медиаторы (ФНО-α, ИЛ-1 и др.), которые, влияя на трансдукцию сигнала, снижают чувствительность клеток-мишеней (прежде всего печени и жировой ткани) к инсулину. Воспаление является непервичным, а дополнительным усиливающим фактором для ИР.

Таким образом, несколько различных реакций на избыточное поступление нутриентов приводят к активному эктопическому накоплению липидов, что вызывает ИР в скелетных мышцах и печени. В данной модели гиперинсулинемия развивается как реакция на ИР, она вторична по отношению к дисфункции митохондрий, и ее роль относительно невелика.

Исходя из описанной концепции, для профилактики ИР (и, соответственно, связанных с ней ВЗЗ) необходимо избегать висцерального ожирения, а для этого поддерживать баланс между энергетической ценностью поступающей в организм пищи и расходом энергии. Это должно предотвратить накопление токсичных полупродуктов окисления, способствовать сохранению нормальной чувствительности к инсулину и, следовательно, поддержанию его выработки на оптимальном уровне. Для устранения уже развившейся ИР необходимы фармакотерапия и превышение затрат энергии над ее поступлением (перейти на «рациональное питание», преимущественно низкокалорийное, в сочетании с «достаточной физической активностью»).

## Модель 2: Инсулинорезистентность как следствие гиперинсулинемии, ожирение — одно из проявлений инсулинорезистентности.

Эта модель патогенеза ИР гораздо менее известна широкой профессиональной аудитории.

Большой вклад в изучение распространенности ГИ в популяции внес американский исследователь Joseph Kraft. В период с 1972 по 1992 гг. он собрал обширную (15 тысяч человек) базу данных профилей выработки инсулина пациентами натощак и через 30, 60, 120 и 180 мин после перорального теста на толерантность к глюкозе (ПТТГ). Впоследствии эта база была тщательно проанализирована [12–14].

Было обнаружено несколько характерных паттернов выработки инсулина:

Основные результаты популяционного исследования.

Среди публикаций, поддерживающих вторую модель, можно выделить несколько ключевых обзоров, в которых детально обсуждаются причинно-следственные связи между ожирением, ИР и ГИ. Описание «углеводно-инсулиновой» модели ожирения иИР представлено ниже как синтез этих обзоров.

История возникновения и конкуренции двух моделей развития ожирения восходит к началу XX в. [15]: эндокринная модель, предложенная еще в 1908 г. выдающимся немецким врачом Von Bergmann и поддержанная другими авторитетными клиницистами, долгое время успешно применялась на практике, в то время как модель энергетического баланса подвергалась жесткой критике [16]. В 1930-е гг. доминировала эндокринная модель, так как ее подкреплял положительный практический опыт [17].

Однако после Второй мировой войны распространение научных идей, изложенных на немецком языке, постепенно сошло на нет, и к 1960-м гг., когда было выявлена ключевая роль инсулина в жировом обмене, уже преобладала модель энергетического баланса, а эндокринная гипотеза была «забыта». К этому времени в англоязычной научной среде ожирение воспринималось преимущественно как нарушение пищевого поведения, и им занимались больше психологи и психиатры [15]. С повестки дня ушла концепция избытка углеводистой пищи, возможной роли избыточной выработки инсулина и связанной с этим ИР, что позволило во многом переложить ответственность за ожирение с системы питания на «обжорство» и «лень» пациента [17].

«Переоткрытие» эндокринной (углеводно-инсулиновой) модели ожирения происходит только в последние 10 лет, по мере накопления убедительных данных о роли ГИ в патогенезе ИР и ожирения.

Современная версия углеводно-инсулиновой модели ожирения была сформулирована в 2018 г. D. Ludwig и C. Ebbeling [18]. В этой работе изложено обоснование и даны ответы на возможные критические вопросы к модели. Так, основной первопричиной развития ожирения служит питание, приводящее к ГИ — а это прежде всего избыток в рационе простых углеводов. В свою очередь, ГИ вызывает отложение жира, снижает доступность энергетических субстратов (жира) из-за угнетения его мобилизации (липолиза) и утилизации (бета-окисления в митохондриях). Меньшая доступность выработки энергии из жиров вызывает энергетический дефицит (проявляющийся в т.ч. мышечной слабостью, усталостью) и повышенный аппетит. То есть переедание является не только и не столько причиной, но и следствием ожирения. Причинно-следственные связи между энергетическим балансом и отложением жира обратны в сравнении с таковыми в модели энергетического баланса: чем больше у человека откладывается жира (на фоне избытка простых углеводов), тем больший энергетический дефицит испытывают его клетки из-за невозможности доступа к накопленным запасам и снижения метаболической пластичности. При этом простое ограничение калорий является стратегически необоснованной симптоматической мерой: низкокалорийная низкожировая диета лишь обостряет проблему энергетического дефицита, запуская режим голодания, снижая скорость обмена и вызывая хронический дистресс [18].

Templeman et al. предложено очень элегантное экспериментальное обоснование ГИ как основной причины ожирения [19]. Авторы исходят из того, что инсулин напрямую модулирует биохимические механизмы поглощения липидов клетками, липолиза и липогенеза. Повышенный уровень инсулина непосредственно связан с ожирением; пищевые и фармакологические воздействия, снижающие уровень инсулина, снижают и массу тела. Однако роль гиперсекреции инсулина как причины развития ожирения оставалась неоднозначной в отсутствие прямых экспериментов с потерей функции. В данной работе такие эксперименты представлены. В частности, у мышей, генетически неспособных к гиперинсулинемии, оказалось невозможным вызвать алиментарное ожирение и жировой гепатоз (ключевое проявление ИР печени). У таких мышей на фоне гиперкалорийного рациона вместо ожирения происходит повышение базального обмена и трансформация белой жировой ткани в бурую.

Существуют и другие убедительные экспериментальные свидетельства ведущей роли ГИ в развитии ИР и ожирения [20][21].

То, что именно повышенная выработка инсулина служит причиной набора массы тела в человеческой популяции, подтвердил, в частности, метод двусторонней менделевской рандомизации [22]: на выборке более 300 тысяч пациентов были сопоставлены данные о генетически обусловленной предрасположенности к повышенной выработке инсулина через 30 мин после ПТТГ и об индексе массы тела (ИМТ). Оказалось, что достоверность взаимосвязи между гиперсекрецией инсулина после ПТТГ и ИМТ настолько высока (β=0,098, P=2,2×10-21), что не оставляет сомнений в причинно-следственной связи между гиперсекрецией инсулина в ответ на глюкозу и избыточной массой тела, то есть подтверждает углеводно-инсулиновую модель ожирения. Напротив, генетическая предрасположенность к высокому ИМТ не влияет на выработку инсулина после ПТТГ.

Тот факт, что гиперсекреция инсулина предшествует инсулинорезистентности и является ее причиной, имеет экспериментальные доказательства и в условиях in vitro [23]. ГИ — это гормональный ответ на избыточное поступление в организм глюкозы и других активаторов бета-клеток ПЖ. Если он сочетается с определенными индикаторами редокс-состояния организма (избытка энергетических субстратов) и АФК, то развивается ИР. В качестве основного индикатора редокс-состояния науровне клетки выступает NAD+ [24] — важнейший редокс-метаболит, косубстрат для работы митохондрий, предиктор эффективности их работы. Он критически важен для регуляции метаболизма и долгожительства, влияет на выработку инсулина и развитие ИР. При старении и нарушении функции митохондрий уровень NAD снижается, и нарушение гомеостаза NAD+ выявляется при всех ВЗЗ, включая нейродегенеративные, СД2 и ЗНО. Поэтому NAD рассматривается как перспективный инструмент лечения нейродегенеративных и метаболических заболеваний [25].

Одним из острых дискуссионных вопросов является соотношение роли углеводов и жиров в липогенезе и индукции ожирения [26]. Для развития ожирения наиболее опасно сочетание в рационе углеводов с высоким гликемическим индексом и жиров. При этом углеводы играют роль «водителя», стимулирующего запасание жиров за счет значительного повышения выработки инсулина. Жир в этом процессе играет пассивную роль («пассажира»): под действием высокого уровня инсулина жировая ткань активно поглощает поступившие с пищей жиры. При этом мобилизация жиров и их утилизация в митохондриях через бета-окисление блокируются инсулином и высоким уровнем глюкозы.

Хроническое повышение выработки инсулина после еды (а еще важнее — натощак) стимулирует липогенез и захват триглицеридов адипоцитами, поэтому играет основную роль в развитии ожирения. Этот процесс особенно важен при метаболическом программировании плода: ГИ и ожирение у беременной женщины «программируют» потомство на развитие ожирения (в раннем возрасте) и метаболических нарушений. Тезис о ключевой роли ГИ (прежде всего связанной с рационом и режимом питания) в развитии ИР у человека подтверждается большим количеством клинических исследований [27–32].

Преобладающую парадигму о первичности ожирения относительно ИР и ГИ ставят под сомнение клинические наблюдения, особенно в случаях, когда подозрение об ИР возникает при необъяснимой ГИ. Базальная ГИ (натощак) часто выявляется до развития ИР, ожирения и/или гипергликемии. Первичная гиперсекреция инсулина связана с худшим метаболическим фенотипом и клиническим прогнозом [33]. Даже в отсутствие ИР повышенная секреция инсулина натощак и после еды вызывает развитие ожирения (у лиц с ожирением и без ИР секреция инсулина повышена более чем на 50%), а снижение массы тела сопровождается снижением секреции инсулина на 35% даже без изменений чувствительности к инсулину [34]. То есть ГИ у лиц с ожирением — это не компенсация ИР, а причина ожирения.

Еще факты: при СД 1 типа введение инсулина часто приводит к системной ИР [35], у детей с ГИ повышен риск развития ожирения во взрослом возрасте, и ГИ является ранним индикатором метаболической дисфункции [36].

Таким образом, ведущая роль ГИ в развитии ожирения и ИР известна более 100 лет, заново «открыта» и подробно описана в современной версии углеводно-инсулиновой модели и подкреплена большим количеством экспериментальных и клинических данных.

Что же позволяет говорить о большом значении ГИ для развития ВЗЗ в целом, а не только метаболических расстройств? Как минимум, это логически вытекает из того, что ожирение признано важнейшим фактором риска практически всех ВЗЗ. А воснове ожирения (гиперплазии и гипертрофии адипоцитов) лежит именно ГИ. Два основных механизма, опосредующие ­причинно-следственные связи между ГИ и ВЗЗ (и даже в целом процессом старения) — это ИР и хроническое воспаление [37][38].

ИР развивается по следующей логике [38].

На фоне ГИ резистентность к инсулину в разных тканях развивается не одновременно. Особый интерес представляет то, что развитие ИР происходит прежде всего в печени, скелетных мышцах и жировой ткани, а также в центральной нервной системе (ЦНС).

В инсулинзависимых тканях на развитие ИР влияют выраженность, длительность ГИ и наличие пауз. А эти факторы, в свою очередь, определяются составом рациона (ИИ пищи), режимом питания, наличием окислительного стресса и стимуляторов активности бета-клеток.

В мышцах ИР усиливается при отсутствии двигательной активности и на фоне высокого уровня кортизола: последний не только вызывает гипергликемию (за счет гликогенолиза и глюконеогенеза), но и напрямую снижает экспрессию GLUT-4, что препятствует поглощению глюкозы мышцами. Все это в целом провоцирует ГИ в сочетании с гипергликемией. Это состояние может развиваться на фоне хронического стресса и приема глюкокортикоидов. Среди лекарственных препаратов, способных вызывать ГИ, помимо кортикостероидов отмечены сульфонилмочевина, антипсихотические препараты и статины.

Ключевая негативная роль фруктозы в развитии ИР печени в последние годы привлекает большое внимание и находит всё большее подтверждение. Кроме того, избыток фруктозы вызывает повышение выработки мочевой кислоты и снижение активности эндотелиальной NO-синтазы, что способствует повышению тонуса сосудов, развитию эндотелиальной дисфункции и артериальной гипертензии [42], а также подагры.

ИР находится в реципрокных отношениях с гипергликемией (ГГ). С одной стороны, ГГ сама по себе вызывает ИР (через ГИ и процессы гликирования); с другой стороны, ГГ может быть обусловлена ИР как в мышцах (снижение утилизации затрудняет снижение уровня глюкозы в крови), так и в печени (например, под действием фруктозы), при которой печень не метаболизирует глюкозу (в гликоген) и не тормозит глюконеогенез — то есть в условиях ИР печень активно вырабатывает глюкозу.

Взаимоотношения и причинно-следственные связи между хроническим системным воспалением, метаболической дисфункцией и ИР во многом остаются открытым вопросом [37]. С помощью противовоспалительных препаратов невозможно устранить ИР. Напротив, хроническая ГИ повышает системную экспрессию провоспалительных цитокинов (за счет прямого действия на иммуноциты) и стимулирует тканеспецифичное воспаление, прежде всего, в жировой ткани. Пролиферативные эффекты инсулина и формирование провоспалительного микроокружения в тканях на фоне ГИ особенно важны для развития ЗНО, и ГИ повышает риск ЗНО независимо от СД2, ожирения и метаболического синдрома [37].

Патофизиологические механизмы влияния ГИ на организм обусловлены многообразием эффектов инсулина на органы и ткани: повышение выработки АФК и конечных продуктов гликирования, стимуляция клеточной пролиферации, истощение идисфункция бета-клеток ПЖ в результате длительной ГИ [43], повышенная выработка жирных кислот и триглицеридов печенью (это основной источник дислипидемии при ИР), повышение экспрессии провоспалительных цитокинов, задержка натрия и воды. Посредством этих механизмов ГИ и прямо, и опосредованно вносит вклад в развитие большинства ВЗЗ и метаболических нарушений, в т.ч. всех хронических воспалительных состояний, метаболического синдрома, атеросклероза и ряда других сердечно-сосудистых заболеваний, гестационного диабета и СД2, неалкогольного жирового гепатоза, ожирения, некоторых видов ЗНО, болезни Альцгеймера и других деменций, глаукомы, подагры, шизофрении и аутизма [38].

## Практические аспекты

Описанные патогенетические модели ожирения (эндокринная и энергетического баланса) предполагают различие подходов к решению проблемы и ожирения, и ИР. Согласно модели энергетического баланса, не важно, в форме каких нутриентов человек получает калории; главное — общее содержание и плотность калорий, вне зависимости от их влияния на выработку гормонов; в лечении главное — это создание дефицита калорий: нужно «меньше есть» (особенно пищи с высоким содержанием калорий — то есть жиров) и «больше двигаться».

Согласно эндокринной модели, ожирение и связанные с ним заболевания обусловлены ГИ, в первую очередь из-за избытка легкоусвояемых углеводов. Это предполагает принципиально иную стратегию профилактики и лечения ожирения: необходимо снижать выработку инсулина через изменение рациона (прежде всего, ограничение продуктов с высоким ИИ для снижения пиковой выработки инсулина) и удлинение интервалов между приемами пищи (для снижения базальной выработки инсулина) [17][18].

Впрочем, обе модели, скорее, взаимно дополняют, чем полностью исключают друг друга: избыток инсулина действительно влияет на пищевое поведение, подавляя насыщение и стимулируя аппетит, а правильное ограничение калорийности рациона (при низкоуглеводной диете) обеспечивает стойкий клинический результат. Основное различие между моделями энергетического баланса и эндокринной состоит в практических выводах: что делать, чтобы остановить прогрессирование ожирения и развитие ИР [15][17].

## ОБСУЖДЕНИЕ

В обзоре суммированы данные последних лет о значении ГИ в развитии ИР. Обсуждаются механизмы взаимосвязей между ГИ, ИР и ожирением, а также некоторые практические подходы к профилактике ГИ, связанные с коррекцией факторов образа жизни. Проанализированы две модели, описывающие патогенез ИР — важнейшего фактора риска и механизма развития ВЗЗ. Графически модели представлены на рисунках 1 и 2.

**Figure:**
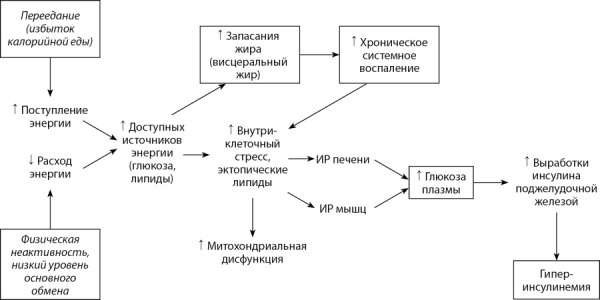
Рис. 1. Общепринятая модель ожирения как нарушения энергетического баланса. В рамки помещены параметры, которые могут быть измерены количественно.Figure 1. The generally accepted model of obesity as an energy imbalance. Boxes contain parameters that can be measured quantitatively.

**Figure:**
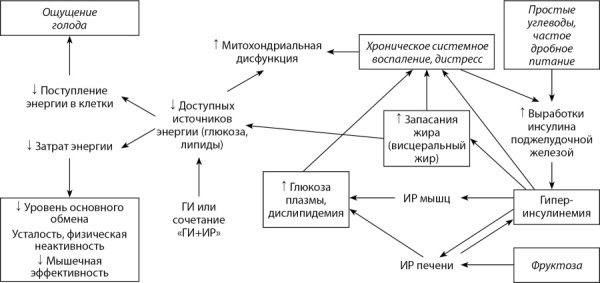
Рис. 2. Углеводно-инсулиновая (эндокринная) модель ожирения. Рамкой обозначены параметры, которые могут быть измерены количественно.Figure 2. Carbohydrate-insulin (endocrine) model of obesity. The box indicates the parameters that can be measured quantitatively.

В медицинском сообществе доминирует модель, согласно которой первичен дисбаланс между поступлением энергии с пищей и ее расходованием. Считается, что такой дисбаланс приводит к ожирению (без уточнения конкретных механизмов), дисфункции митохондрий и системному воспалению, которые и служат причиной ИР. В свою очередь, в качестве компенсаторной реакции на ИР развивается ГИ. В такой модели стратегия борьбы с ИР основана на двух подходах: 1) снижении избыточной массы тела через низкокалорийную диету с последующим переходом на изокалорийную диету, в сочетании с повышением физической активности; 2) прямом снижении ИР посредством фармакотерапии. Таким образом, устранение ИР и снижение избыточной массы тела предлагается осуществлять в основном в рамках парадигмы энергетического баланса [16]. Как основа для долгосрочной коррекции избыточной массы тела и профилактики ВЗЗ эта модель продемонстрировала свою низкую ­результативность: в последние 30 лет во всем мире продолжается неуклонный рост ожирения, заболеваемости и смертности от ВЗЗ — фактический «дефолт» системы профилактики ВЗЗ.

Преобладающая модель во многом игнорирует не только большой массив экспериментальных и клинических данных, указывающих на иные причинно-следственные связи между ГИ, ИР и ожирением, но и канонические представления о ведущей физиологической роли инсулина в липогенезе, липолизе и утилизации жиров — то есть в развитии ожирения. В том числе из-за этого на основе некорректных представлений формируется заведомо неэффективная стратегия борьбы с ожирением, ИР и в конечном счете — с ВЗЗ.

Вторая модель патогенеза ИР является не чем-то принципиально новым, а логическим продолжением и развитием эндокринной модели ожирения, сформулированной еще в начале ХХ в. Эта модель не несет глубинного противоречия с моделью энергетического баланса, а уточняет ее. Она в полной мере учитывает современные представления о молекулярных механизмах действия инсулина, о ведущей роли митохондрий в энергетическом обмене и о естественном чередовании основных энергетических субстратов в клетке. Так, в период после приема пищи преимущественным субстратом окисления является глюкоза, а в состоянии пищевой паузы — жиры. Инсулин служит одним из ключевых переключателей между этими режимами, поэтому при хронической ГИ происходит снижение метаболической гибкости, необходимой для адаптации [44].

Борьба с ИР, основанная на снижении массы тела через ограничение калорийности рациона, оказывается эффективной лишь при правильном подборе состава этого рациона и режима питания. В частности, низкожировая диета значительно уступает по долгосрочной эффективности низкоуглеводной диете [45], и в целом лишь менее 20% пациентов, снизивших массу тела на низкокалорийной диете, сохраняют полученный результат в течение длительного времени [46]. Таким образом, борьба с ожирением и ИР в реальной клинической практике остается нерешенной проблемой [47], что в целом указывает на несостоятельность доминирующей модели ИР.

ГИ можно рассматривать как самый ранний симптом и причину метаболических нарушений, в т.ч. метаболического синдрома. Важно отметить, что ГИ натощак предшествует развитию гипергликемии в среднем на 20–24 года [43][48][49]. ГИ предшествует развитию ожирения и является фактором его развития [50][51]. Наиболее ранним точным маркером предиабета, СД2 и повышенного сердечно-сосудистого риска является не отдельно ПТТГ или уровень инсулина натощак, а профиль выработки инсулина (наличие ГИ) после ПТТГ [14].

## ЗАКЛЮЧЕНИЕ

ИР служит ранним универсальным предиктором важнейших ВЗЗ, поэтому полнота и адекватность модели патогенеза ИР критически важны для выстраивания эффективной стратегии борьбы с ВЗЗ.

Преобладающая сегодня модель развития ИР (согласно которой первично ожирение, а ГИ развивается как реакция на ИР) вступает в противоречие с большим массивом экспериментальных и клинических данных. Основанная на ней стратегия борьбы с ВЗЗ (акцент на низкокалорийной диете и фармакологической коррекции ИР) в целом показала недостаточную результативность.

Современным системным представлениям о роли инсулина в энергетическом и пластическом обмене, массиву экспериментальных и клинических данных в большей степени соответствует альтернативная модель патогенеза ИР, согласно которой первична хроническая ГИ, а ИР и ожирение в целом вторичны.

Стратегия борьбы с ВЗЗ, вытекающая из альтернативной модели (акцент на низкоуглеводном рационе и достаточной пищевой паузе в сочетании с учетом других факторов развития ИР), открывает возможности для ранней (донозологической) профилактики широкого спектра хронических заболеваний, в патогенезе которых значима роль ИР.

## ДОПОЛНИТЕЛЬНАЯ ИНФОРМАЦИЯ

Источники финансирования. Аналитическое исследование выполнено в рамках государственного задания № 075-00483-21-01 «Превентивные технологии персонализированной геропротекции».

Конфликт интересов. Авторы декларируют отсутствие явных и потенциальных конфликтов интересов, связанных с содержанием настоящей статьи.

Участие авторов. А.В.Матюшев-Поклад — концепция работы, получение, анализ данных, написание статьи. Д.С.Янкевич — концепция работы, анализ данных, интерпретация результатов, внесение в рукопись существенных правок. М.В.Петрова — концепция работы, внесение в рукопись существенных правок. Н.Г.Савицкая — концепция работы, внесение в рукопись существенных правок. Все авторы одобрили финальную версию статьи перед публикацией, выразили согласие нести ответственность за все аспекты работы, подразумевающую надлежащее изучение и решение вопросов, связанных с точностью или добросовестностью любой части работы.
